# Retraction Note: Deregulated AJAP1/*β*-catenin/ZEB1 signaling promotes hepatocellular carcinoma carcinogenesis and metastasis

**DOI:** 10.1038/s41419-024-06788-2

**Published:** 2024-06-05

**Authors:** Jihua Han, Changming Xie, Tiemin Pei, Jiabei Wang, Yaliang Lan, Kaihua Huang, Yifeng Cui, Fengyue Wang, Jiewu Zhang, Shangha Pan, Yingjian Liang, Tongsen Zhen, Ruipeng Song, Boshi Sun, Yuejin Li, Huawen Shi, Guangchao Yang, Xirui Liu, Mingxi Zhu, Yan Wang, Keyu Li, Yao Liu, Fanzheng Meng, Fei Liao, Xianzhi Meng, Xuehui Hong, Lianxin Liu

**Affiliations:** 1 Key Laboratory of Hepatosplenic Surgery, Ministry of Education, Department of General Surgery, the First Affiliated Hospital of Harbin Medical University, Harbin, China; 2 Department of Head and Neck Surgery, The Third Affiliated Hospital of Harbin Medical University, Harbin, China; 3 Department of Gastrointestinal Surgery, Zhongshan Hospital of Xiamen University, Xiamen, China; 4 Institute of Gastrointestinal Oncology, Medical College of Xiamen University, Xiamen, China

Retraction to: *Cell Death and Disease* 10.1038/cddis.2017.126, published online 06 April 2017

The Editors-in-Chief have retracted this article after concerns were raised about the data reported. Specifically:

- In Figure 3B, the top SMMC7721 panels appear to overlap. The bottom shCon panel of SMMC7721 panel of this figure also appears to overlap with the shZEB1 migration panel of SMMC77210shAJAP1 in Figure 6A.
- The AJAP1 panel of HCCLM3 in Fig. 3E appears to overlap with the shZEB1 panel of SMMC7721-shAJAP1 in Figure 6B.
- In Figure 6A, there appears to be an area of overlap between the top shAJAP1 panel of SMMC7721 and the top shCon panel of SMMC7721-shAJAP1.

The Editors-in-Chief no longer have confidence in the results and conclusions of this article.

Lianxin Liu and Jihua Han disagree with this retraction. The Publisher was not able to obtain a current email address for Shangha Pan. The remaining authors did not respond to correspondence from the publisher about this retraction.

